# Bone marrow adipocytes and lung cancer bone metastasis: unraveling the role of adipokines in the tumor microenvironment

**DOI:** 10.3389/fonc.2024.1360471

**Published:** 2024-03-20

**Authors:** Jian Li, Jialu Wu, Yanni Xie, Xijie Yu

**Affiliations:** ^1^ Laboratory of Endocrinology and Metabolism/Department of Endocrinology and Metabolism, Rare Disease Center, West China Hospital, Sichuan University, Chengdu, China; ^2^ Department of Endocrinology and Metabolism, Shandong Second Provincial General Hospital, Jinan, China

**Keywords:** bone marrow adipocytes, adipokines, tumor bone metastasis, lung cancer bone metastasis, immune response

## Abstract

Bone is a common site of metastasis for lung cancer. The “seed and soil” hypothesis suggests that the bone marrow microenvironment (“soil”) may provide a conducive survival environment for metastasizing tumor cells (“seeds”). The bone marrow microenvironment, comprising a complex array of cells, includes bone marrow adipocytes (BMAs), which constitute about 70% of the adult bone marrow volume and may play a significant role in tumor bone metastasis. BMAs can directly provide energy for tumor cells, promoting their proliferation and migration. Furthermore, BMAs participate in the tumor microenvironment’s osteogenesis regulation, osteoclast(OC) regulation, and immune response through the secretion of adipokines, cytokines, and inflammatory factors. However, the precise mechanisms of BMAs in lung cancer bone metastasis remain largely unclear. This review primarily explores the role of BMAs and their secreted adipokines (leptin, adiponectin, Nesfatin-1, Resistin, chemerin, visfatin) in lung cancer bone metastasis, aiming to provide new insights into the mechanisms and clinical treatment of lung cancer bone metastasis.

## 1 Introduction

According to data released by the Global Burden of Disease Study in 2020, an estimated 2.2 million people worldwide are afflicted with lung cancer, with approximately 1.8 million deaths. As the cancer with the highest incidence and mortality rate globally, lung cancer is the leading cause of cancer death among men and the second leading cause among women. Despite substantial regional variations in incidence and mortality rates between men and women, statistics show that rates in men are approximately twice those in women (1). Compared to other malignancies, lung cancer continues to have a low survival rate, with a 5-year survival rate of only 5% for advanced stages (2). One of the main characteristics of malignant tumors is their ability to metastasize, and over 90% of lung cancer patients die from complications related to metastasis. Bone is among the most common metastasis sites for lung cancer, with an incidence rate of 30-40%. Clinical data indicate that approximately 40-48% of patients with advanced lung cancer exhibit bone metastasis at initial diagnosis (3). Once bone metastasis occurs, the median survival time of patients significantly reduces to only five months (3).Depending on radiological features, lung cancer bone metastasis can be classified into osteolytic, osteoblastic, and mixed types. Studies have shown that approximately 70% of lung cancer bone metastases are osteolytic, with osteoblastic types being less common (4). Following osteolytic bone metastasis in lung cancer, about 50% of patients will experience skeletal-related events (SREs), including intractable bone pain, pathological fractures, spinal cord compression, and hypercalcemia, which accelerate disease deterioration, diminish quality of life, decrease physical abilities, increase medical expenses, and elevate mortality rates. Given that current treatments for lung cancer bone metastasis are limited to symptom relief and cannot effectively slow the progression or fundamentally alter the pathological process, elucidating the pathogenic mechanisms of lung cancer bone metastasis and exploring effective early diagnosis and treatment strategies are crucial.

## 2 Pathological processes of lung cancer bone metastasis

**Figure 1:**
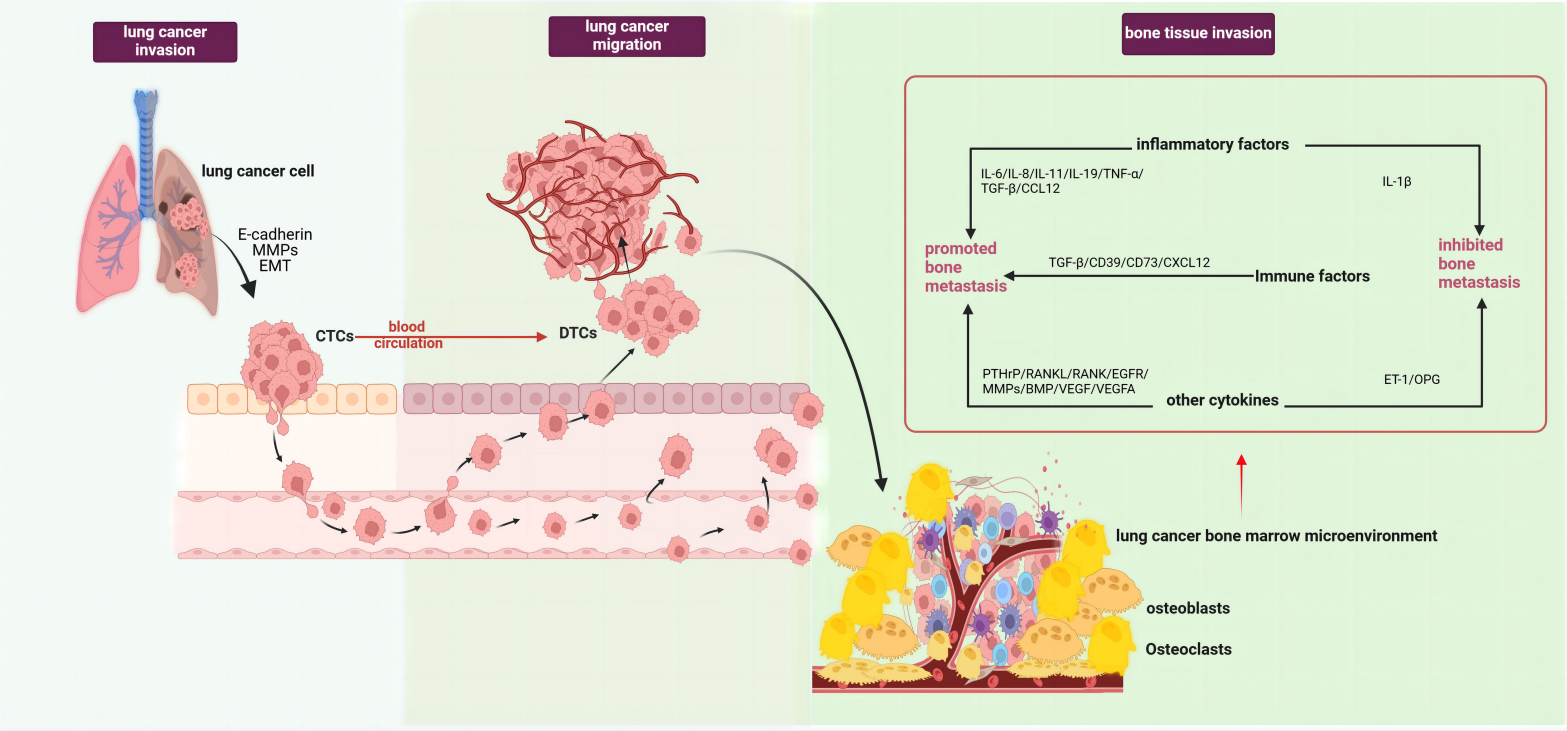
Pathological processes of lung cancer bone metastasis (created with BioRender.com.). The occurrence of bone metastasis in lung cancer could be divided into three stages, tumor invasion, tumor cell migration, and bone tissue invasion. In the first stage, lung cancer cells secreted E-cadherin and MMPs, along with EMT of lung cancer cells, enabling their entry into circulation and formation of DTCs. In the second stage, lung cancer cells detached from the tumor matrix and entered circulation to become CTCs. In the third stage, after lung cancer cells migrated to the bone marrow microenvironment, under the combined effects of various interactions between different cell types and factors within the tumor microenvironment, they became reactivated and formed proliferative metastatic lesions. This stage mainly involved the roles of various factors in the tumor microenvironment in bone metastasis,such as pro-metastatic inflammatory factors (IL-6, IL-8, IL-11, IL-19, TNF-α, CCL12), immune factors (TGF-β, CD39, CD73, CXCL12), and other factors (PTHrP, MMPs, RANKL, RANK, BMP, EGFR/VEGF, VEGFA). Factors that inhibited lung cancer bone metastasis included IL-1β, OPG, and ET-1. EMT, epithelial-mesenchymal transition; MMPs, matrix metalloproteinases; DTCs, Disseminated tumor cells; CTCs, Circulating tumor cells; IL-1β, Interleukin 1 beta; IL-6, Interleukin 6; IL-8, Interleukin 8; IL-11, Interleukin 11, IL-19, Interleukin 19; CCL12, C-C motif chemokine ligand 12; TNF-α, Tumor necrosis factor alpha; TGF-β, Transforming growth factor beta; CD39, Cluster of differentiation 39; CD73, Cluster of differentiation 73; CXCL12, C-X-C motif chemokine ligand 12; PTHrP, Parathyroid hormone-related protein; MMPs, Matrix metalloproteinases; RANKL, Receptor activator of nuclear factor kappa B ligand; RANK, Receptor activator of nuclear factor kappa B; BMP, Bone morphogenetic protein; OPG, Osteoprotegerin; ET-1, Endothelin 1.

The occurrence of lung cancer bone metastasis results from a series of complex pathological processes, which can generally be divided into tumor invasion, tumor cell migration, and bone tissue invasion stages. The initiation phase of bone metastasis involves tumor cells escaping the primary site and entering the circulation, forming disseminated tumor cells (DTCs) (5). The epithelial-mesenchymal transition (EMT) plays a key role in this process, where epithelial cells lose polarity and cell-cell adhesion, acquiring mesenchymal characteristics (6). Studies have demonstrated that abnormal activation of the Wnt/β-catenin pathway can induce the onset and progression of tumor EMT (7). Both *in vitro* and *in vivo* studies have observed an increase in β-catenin expression under hypoxic conditions, promoting EMT progression, affecting lung cancer cell migration capabilities, and inducing morphological changes (8). Lung cancer cells can also secrete E-cadherin and matrix metalloproteinases(MMPs), degrading the extracellular matrix, reducing cell adhesion and cross-linking, facilitating tumor cell detachment from the tumor matrix into the circulation as circulating tumor cells (CTCs), and ultimately forming DTCs that migrate to the bone marrow microenvironment (9). Upon reaching the bone marrow microenvironment, tumor cells undergo stages of settlement, survival, and dormancy, eventually reactivating and forming proliferative metastatic foci (5) (**Figure 1**).

## 3 The role of the bone marrow microenvironment in lung cancer bone metastasis

The “seed and soil” hypothesis suggests that during the process of bone metastasis, the bone marrow microenvironment (“soil”) may provide an “ecological niche” suitable for metastasis, laying the foundation for the seeding, invasion, and proliferation of tumor cells (“seeds”) (10). A conducive “soil” may determine whether the “seeds” will germinate, as research has found that less than 0.01% of circulating tumor cells ultimately form distal metastases (11). The activation process of tumor cells within the bone marrow microenvironment (the germination process of “seeds” in the “soil”) is the result of bilateral interaction.

On one hand, tumor cells interact with various cells in the bone microenvironment, such as bone marrow stromal cells (BMSCs), osteoclast (OCs), osteoblasts (OBs), endothelial cells (ECs), BMAs, immune cells, etc., causing adaptive changes in the bone marrow microenvironment and providing favorable conditions for tumor cell invasion and growth (12). For instance, *in vivo* studies have found that BMSCs can be chemotactically guided to tumor cells, participating in the construction of the tumor microenvironment (13). Lung cancer cells can also activate OCs, causing bone matrix dissolution and creating conditions for their adhesion and settlement (14). ECs in the microenvironment can enhance tumor metastasis through angiogenesis, providing an energy source and new pathways for tumor cell invasion and migration (15).

On the other hand, various growth factors secreted by lung cancer cells, such as EGF-like domain multiple 6, bone morphogenetic protein-7 (BMP-7), transforming growth factor-beta (TGF-β), endothelin-1 (ET-1), fibroblast growth factors (FGFs), platelet-derived growth factors (PDGF), etc., can directly affect the composition of the tumor microenvironment (16–19). For instance, EGF-like domain multiple 6 secreted by lung adenocarcinoma cells can enhance the EMT process, activate the Wnt/β-catenin and PI3K/AKT/mTOR pathways, promoting lung adenocarcinoma cell proliferation, migration, and invasion capabilities. Overexpression of this factor in a nude mouse model can enhance tumor growth and exacerbate bone resorption. *In vitro* studies have also found that it can increase OC differentiation of mouse bone marrow mononuclear macrophages via the NF-κB and c-Fos/NFATc1 signaling pathways (16). *In vitro* cell studies have shown that downregulating BMP-7 expression can significantly inhibit the invasiveness of lung adenocarcinoma SPC-A1 cells, while upregulating BMP-7 notably promotes the migration ability of A549 cells (17). Clinical studies have also found BMP-7 expression in the tumor cell membrane and cytoplasm of non-small cell lung cancer (NSCLC), with high cytoplasmic BMP-7 expression associated with tumor progression and adverse prognosis. These results all demonstrate that BMP-7 secreted by lung cancer cells, through affecting cell invasiveness and migration capability, promotes its growth and spread in bone tissues (18). *In vitro* cell studies indicate that TGF-β secreted by lung cancer cells can not only promote tumor microenvironment angiogenesis to facilitate lung cancer cell proliferation and migration but also regulate T cell activity to inhibit the immune system’s recognition and attack on tumor cells, helping the tumor evade immune surveillance (19). Additionally, TGF-β can increase the invasive and migratory capabilities of lung cancer cells, thereby promoting bone metastasis (20). Both *in vivo* and *in vitro* studies have confirmed that MMPs and urokinase plasminogen activator secreted by lung cancer cells can specifically degrade bone matrix components (such as collagen and trabeculae), leading to bone tissue destruction and dissolution, facilitating tumor cell invasion into bone tissues (21). A study on microRNA-328 (miR-328) secreted by lung adenocarcinoma A549 cells discovered that miR-328, potentially through the downregulation of neuropilin-2 (Nrp-2) expression in A549-derived extracellular vesicles (A549-Exos) *in vitro*, enhanced OC formation and bone resorption. Meanwhile, the *in vivo* administration of a miR-328 inhibitor in A549-Exos significantly inhibited bone resorption (22). Lastly, the unique invasiveness, infiltrative capability, and rapid growth and migration ability of tumor cells also affect the transformation of the metastatic foci’s microenvironment (23). Tumor cells can also engage in inflammatory reactions and a series of complex interactions with immune cells in the microenvironment, enabling tumor cells to evade immune surveillance, thereby promoting their proliferation and migration (24). Additionally, the metabolic decomposition of BMAs can produce a large amount of lipids, providing energy for tumor cells, promoting their proliferation, migration, and invasion. BMAs can also secrete certain adipokines, such as leptin, by activating signaling pathways such as PI3K, HIF, and MAPK, enhancing the proliferation, migration, and invasion capabilities of lung cancer cells (25, 26) (**Figure 1**).

### 3.1 The role of OCs in lung cancer bone metastasis

**Figure 2:**
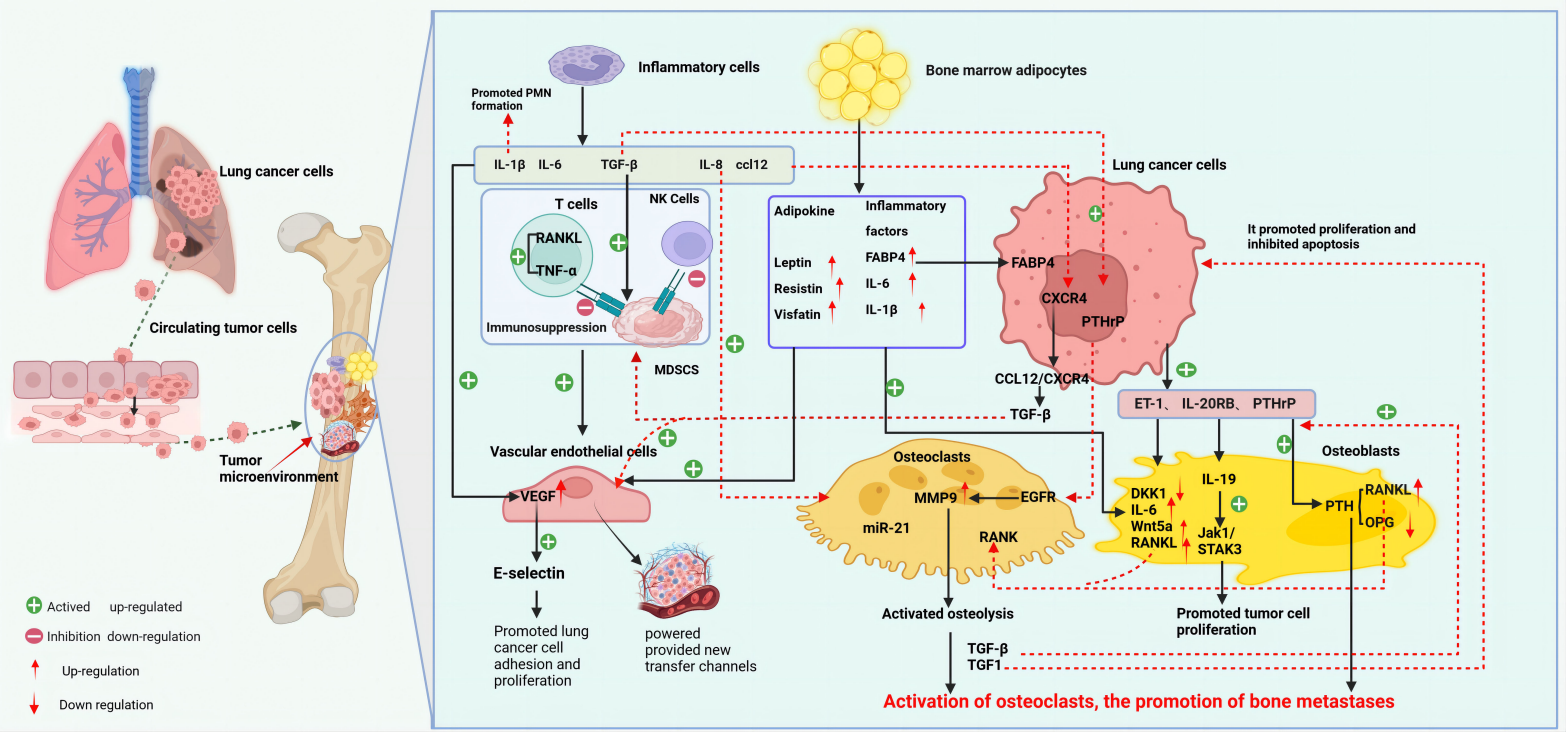
Mechanism of bone metastasis in lung cancer (created with BioRender.com.). The main mechanism of bone metastasis in lung cancer is the interaction between various cells (OC, OB, EC, BMA, inflammatory cells, etc.) in the bone microenvironment and lung cancer cells, which leads to adaptive changes in the bone marrow microenvironment for tumor cell invasion and growth. At the same time, various factors secreted by lung cancer cells also participate in and affect the composition of the tumor microenvironment. Bone metastasis in lung cancer mainly manifests as osteolytic lesions. Lung cancer cells can directly or indirectly activate OC, initiating a vicious cycle of bone resorption, promoting bone matrix dissolution and lung cancer bone metastasis. OB metastasis is relatively rare in lung cancer, mainly through the downregulation of DKK1 in OB by ET-1 secreted by lung cancer cells, upregulation of osteogenic genes such as IL-6 and RANKL, and promotion of OB metastasis. Lung cancer cells, inflammatory cells, and BMA in the bone tumor microenvironment release inflammatory factors, participate in the construction of an immune-suppressive and tumor angiogenesis microenvironment, and provide conditions for lung cancer cells to evade immune surveillance, proliferate,and migrate. BMA, bone marrow adipocytes; ccl12, chemokine 12; CXCR4, CXC chemokine receptor 4; DKK1, Dickkopf-1; ET-1, endothelin-1; EC, endothelial cells; EGFR, epidermal growth factor receptor; IL-19, Interleukin-19; IL-20R, interleukin 20 receptor; MDSCs, myeloid-derived suppressor cells; MMP9, matrix metalloproteinase 9; OB, osteoblasts; OC, osteoclasts; PTHrP, parathyroid hormone-related protein; PTH, parathyroid hormone; RANK, receptor activator of nuclear factor κ B; RANKL, RANK ligand; sVCAM1, soluble vascular cell adhesion molecule-1.

The osteolytic lesions in lung cancer bone metastasis are primarily caused by the activation of OCs. Lung cancer cells can directly influence OCs or indirectly upregulate their function through secreted active factors, thereby promoting lung cancer bone metastasis (27). For instance, lung cancer cells secrete extracellular vesicles containing amphiregulin (AREG), which induces abnormal activation of the EGFR signaling in OCs, upregulating MMP-9 and thus triggering osteolytic metastasis (28). Lung adenocarcinoma secreted miR-21 suppresses programmed cell death 4 (PDCD4), promoting the generation of OCs and hence facilitating osteolytic lesions in lung cancer (29). Additionally, experimental studies with lung cancer cells have found that they secrete parathyroid hormone-related protein (PTHrP), which binds to the PTH/PTHrP receptor, enhances the expression of the receptor activator of nuclear factor-kappa B ligand (RANKL), and inhibits the synthesis of osteoprotegerin (OPG) (30). RANKL binds to the receptor activator of nuclear factor κB (RANK) on the surface of OC precursor cells, inducing OC aggregation and activation, enhancing their activity, and causing osteolytic destruction (31). The release of insulin-like growth factor-1 (IGF-1) and TGF-β from bone matrix, in turn, acts on tumor cells: IGF-1 promotes tumor cell proliferation and inhibits apoptosis, while TGF-β induces the tumor to secrete more PTHrP, activating more OCs, and dissolving the bone matrix again (32), forming a “vicious cycle” that promotes the occurrence and development of tumor bone metastasis. Furthermore, *in vivo* and *in vitro* studies have discovered that lung adenocarcinoma A549 cells induce OCs to secrete the ligand IL-19 for IL-20RB, activating the downstream JAK1/STAT3 signaling pathway, and promoting lung cancer proliferation in the bone microenvironment (33). Importantly, blocking IL-20RB with neutralizing antibodies can significantly inhibit lung cancer bone metastasis. These data demonstrate the important role played by activated OCs in osteolytic bone metastasis of lung cancer (**Figure 2**).

### 3.2 The role of OBs in lung cancer bone metastasis

Compared to osteolytic metastasis, osteoblastic (bone-forming) metastasis of lung cancer is less common (34). Currently, most studies on osteoblastic metastasis suggest that under various influences, OBs participate in tumor bone metastasis through secreting molecules to affect OC formation and tumor progression. Factors promoting osteogenic differentiation of OBs include BMP, ET-1, semaphorin 3A (Sema3A), vascular endothelial growth factor (VEGF), monocyte chemoattractant protein-1 (MCP-1), and interleukin-6 (IL-6), etc. (34–36). For example, Wnt/β-catenin signaling and DKK1 (a Wnt signaling pathway inhibitor) can promote lung cancer metastasis, particularly bone metastasis (37). *In vitro* cell studies discovered that ET-1 significantly downregulates DKK1 in OBs, while also upregulates osteogenic genes (such as IL-6, Wnt5a, RANKL, etc.) (38). Thus, ET-1, as a key tumor factor, can not only upregulate genes promoting osteogenesis but also downregulate negative regulators of osteogenesis, participating in tumor osteoblastic metastasis. Additionally, miR-139-5p was found to positively regulate the osteogenic differentiation of mesenchymal stem cells (MSCs) (39). However, Feng et al. found that miR-139-5p inhibits osteogenic differentiation. *In vivo* model studies showed that upregulation of miR-139-5p reduced cell proliferation and osteogenic differentiation in MSCs by targeting NOTCH1 and inhibiting the Wnt/β-catenin signaling pathway (40). *In vitro* cell studies also found that hypoxia-inducible factor-1α (HIF-1α) can inhibit osteogenic differentiation by upregulating Sema4D, thus participating in lung cancer bone metastasis (41). Engblom et al. found that OBs also have a distal regulatory role in lung cancer progression (42). However, the specific mechanisms of OBs in lung cancer bone metastasis are still not fully understood and require further in-depth research (**Figure 2**).

### 3.3 The role of inflammatory cells in bone metastasis of lung cancer

It is well-documented that inflammation plays a critical role in tumor progression. Both lung cancer cells and inflammatory cells secrete pro-inflammatory cytokines, which not only directly participate in the formation of the pre-metastatic niche (PMN) but also engage in the activation, proliferation, and migration of tumor cells in the microenvironment, thereby facilitating bone metastasis (43). Evidence suggests that cytokines such as interleukin-1β (IL-1β), IL-6, interleukin-7 (IL-7), interleukin-8 (IL-8), interleukin-11 (IL-11), tumor necrosis factor-alpha (TNF-α), TGF-β, and C-C motif chemokine ligand 12 (CCL12) play significant roles in the bone metastasis of lung cancer (43–46). For instance, studies have shown that blocking IL-1β in K-ras mutant lung adenocarcinoma (KM-LUAD) mice, which express high levels of IL-1β in the lungs, significantly reduces tumor load when IL-1β monoclonal antibodies are administered at 6 and 14 weeks of age (47). Clinical research also confirms that inhibiting IL-1β significantly reduces the incidence rate of lung cancer in a dose-dependent manner (48). Lung cancer cells secrete IL-6 and IL-11, which can activate various signaling pathways such as PI3K/Akt and MAPK, promoting cell proliferation and migration (49–52). *In vitro* studies reveal that IL-11 can stimulate OC formation and activation, accelerate bone resorption, and release growth factors such as TGF-β in the bone matrix, thereby promoting the growth and metastasis of lung cancer cells (53). Additionally, IL-8 has been found to increase the invasive capabilities of tumor cells and promote OC maturation and activation, leading to the release of key enzymes such as acid phosphatase and MMPs that degrade the bone matrix and further enhance bone dissolution (54). IL-8 also induces BMSC to secrete RANKL while inhibiting OPG secretion, leading to RANKL/OPG dysregulation, thus promoting OC activation and maturation and subsequent bone resorption (55). Moreover, IL-1, IL-6, IL-8, TNF-α, and TGF-β secreted by lung cancer cells can activate downstream signaling pathways to increase VEGF expression, thereby promoting angiogenesis in the bone tumor microenvironment and indirectly facilitating bone metastasis (56). These inflammatory cytokines can also recruit and induce immune cells, notably myeloid-derived suppressor cells (MDSCs), tumor-associated macrophages, and neutrophils, creating an immunosuppressive local milieu that promotes tumor cell survival and thereby indirectly facilitating bone metastasis (57). *In vitro* cell studies have shown that activation of the KRAS signaling pathway can upregulate tumor cell secretion of CCL12, which promotes tumor cell recruitment to target organs through binding to its receptor CXCR4 (46). Research has confirmed that upregulation of CXCL12 promotes cancer cell metastasis and growth in bone metastases of breast and prostate cancers (58, 59). The expression of CXCR4 is elevated in bone destruction areas of NSCLC bone metastasis patients (60). Furthermore, clinical studies have found TGF-β promoting CD39 and CD73 expression on MDSCs, which can suppress T cell and NK cell activity, thus contributing to the formation of an immunosuppressive microenvironment and enabling tumor cells to evade immune surveillance (61). These findings underscore the pivotal role of inflammatory cells and their secreted cytokines in lung cancer bone metastasis, though the complex inflammatory response mechanisms involved in lung cancer bone metastasis require further research (**Figure 2**).

### 3.4 The role of the RANK/RANKL signaling axis in bone metastasis of lung cancer

The RANK/RANKL signaling axis, crucial for maintaining bone homeostasis, plays a key role in tumor bone metastasis. RANKL, a critical regulator of OC differentiation, is chiefly secreted by OBs, osteocytes, and activated T cells. Binding of RANKL to RANK activates signaling pathways in OC precursors, promoting OC formation and enhancing bone resorption (62). OPG, a competitive receptor for RANKL, inhibits the RANKL-RANK interaction, thereby reducing OC formation and activity (63).

#### 3.4.1 Direct effects of the RANKL/RANK signaling pathway on bone metastasis of lung cancer

The RANKL/RANK signaling pathway directly affects the functionality of lung cancer cells and OCs, playing a role in bone metastasis of lung cancer. *In vitro* studies have revealed that RANKL, by activating the RANK receptor, can promote lung cancer cell proliferation and growth while inhibiting apoptosis (64). Other research has shown that activation of the RANKL/RANK signaling pathway enhances OC functionality within the bone marrow microenvironment, aiding bone resorption and consequently facilitating bone metastasis (65).

#### 3.4.2 Indirect effects of the RANKL/RANK signaling pathway on bone metastasis of lung cancer

The indirect mechanisms in bone metastasis include: 1) Activation of NF-κB, MAPK, and other signaling pathways to promote lung cancer proliferation and growth (66). 2) Release of inflammatory cytokines and other cellular factors, altering the composition of the tumor microenvironment and indirectly facilitating bone metastasis (62). 3) Regulation of the activation and function of immune cells within the tumor microenvironment. In certain cases, activation of the RANKL/RANK signaling pathway induces immunosuppressive molecules (e.g., TGF-β from Treg cells), regulating the immune response balance, allowing lung cancer cells to evade immune surveillance and promote bone metastasis (67). Moreover, RANKL can stimulate the maturation and activation of dendritic cells, macrophages, and other immune cells, enhancing their cytokine and chemokine production, thus boosting antigen presentation and T cell activation capabilities (65, 68). Additionally, RANKL/RANK signaling activation can regulate the NF-κB signaling pathway to inhibit B cell apoptosis, promoting B cell survival and proliferation capacity, providing a protective environment for lung cancer cells to escape immune surveillance, thereby inhibiting lung cancer cell apoptosis and promoting bone metastasis (69). In summary, RANKL/RANK maintains immune homeostasis through various immune regulation mechanisms, indirectly participating in bone metastasis. 4) Regulation of VEGF receptor activation and tumor angiogenesis. It also promotes EC growth, migration, and lumen formation, contributing to tumor microenvironment angiogenesis (70, 71). The RANK/RANKL signaling axis plays a complex role in lung cancer bone metastasis, with the specific mechanisms requiring further in-depth study.

### 3.5 The role of lung cancer cell apoptosis in bone metastasis

Apoptosis functions as a critical protective mechanism within organisms, which eliminates aberrant cells and prevents tumor genesis. When apoptosis of tumor cells is inhibited, cells may evade immune surveillance, thereby enhancing their survival and propagation (72). *In vitro* studies have revealed that lung cancer cells can suppress the initiation of apoptosis by upregulating the anti-apoptotic protein Bcl-2, which blocks the release of apoptogenic cytochrome c; they can also inhibit apoptosis by downregulating pro-apoptotic proteins, such as Bax and Bak, thus promoting their survival within the bone marrow microenvironment (73, 74). Furthermore, lung cancer cells can interact with other cells in the bone microenvironment (such as OBs, OCs, and BMAs), not only augmenting the anti-apoptotic capabilities of lung cancer cells but also enhancing the function of OCs (73). For instance, proteins of the Bcl-2 family can inhibit the apoptosis of lung cancer cells and promote the differentiation and function of OCs. Cytokines released during the apoptosis of lung cancer cells, such as TNF-α, TGF-β, and VEGF, can indirectly promote bone metastasis by fostering inflammation and angiogenesis within the tumor microenvironment (75). Additionally, the release of lactate and ATP post-apoptosis can upregulate the metabolic state of bone cells and increase acidification of the tumor microenvironment, indirectly facilitating bone resorption in the context of lung cancer bone metastasis (76).

### 3.6 Mechanisms of angiogenesis and VEGF in bone metastasis

During tumor metastasis, lung cancer cells and ECs release VEGF to promote the formation of new blood vessels, supplying the tumor with additional nutrients and growth factors, and providing new physical pathways for tumor metastasis. For example, MDSCs, as critical molecules in PMN formation, can produce VEGFA, which upregulates E-selectin, thereby enhancing the adhesion of tumor cells, and facilitating the homing and proliferation of circulating tumor cells (77). Furthermore, MDSCs can secrete MMP9 to regulate the function of VEGF to promote angiogenesis and tumor cell extravasation and migration (78, 79). Increasing research indicates that adipokines (such as leptin, resistin, visfatin, etc.) and inflammatory factors (such as IL-1β, IL-6, chemokines, FABP4, etc.) secreted by BMA can also regulate angiogenesis, indirectly facilitating the progression of tumor bone metastasis (80–83). Occupying 70% of the bone marrow cavity volume, BMAs constitute a major component of the bone marrow microenvironment, and their role in tumor bone metastasis is gaining increasing attention, particularly the regulatory effects of secreted adipokines on energy metabolism, endocrine functions, and inflammatory responses in influencing tumor growth and migration (81, 83). Despite numerous studies on tumor bone metastasis and the bone marrow microenvironment, the mechanisms linking BMAs and lung cancer bone metastasis remain largely unexplored. This text will next focus on elucidating the mechanisms underlying the role of BMAs and their secreted adipokines in lung cancer bone metastasis (**Figure 2**).

## 4 The role of BMAs in lung cancer bone metastasis

### 4.1 Origin and distribution of BMAs

BMAs originate from a distinct cell population within the bone marrow, comprising MSCs and preadipocytes of the marrow adipogenic lineage (84). While historically regarded as an inert adipose tissue, recent studies have identified unique characteristics and functions of BMAs, distinguishing them from white, brown, and beige adipose tissues (85, 86). In humans, BMAs are primarily located in the marrow of long bones, especially within the trabecular bone at the epiphyses and metaphyses, and near the endosteal surface of the bone shaft (87). The abundance of BMAs in the bone marrow increases with aging, obesity, the application of peroxisome proliferator-activated receptor γ (PPARγ), and radiation exposure (85, 88). Studies in ovariectomized mouse models have shown that estrogen deficiency leads to an increase in BMAs, which can be reversed by estrogen supplementation (89). Clinical research on osteoporosis has revealed that the age-related increase in BMAs is associated with bone loss, suggesting BMAs as negative regulators of bone mass (90). However, this correlation is not uniformly observed across studies. In C57BL/6 mice (with the lowest trabecular and cortical bone density among all mouse strains), BMAs are scarce, whereas they are abundant in C3H/He mice (a unique strain with higher bone density) (91, 92), implying genetic regulation of BMA distribution and the need for further comprehensive analysis (**Figure 2**).

### 4.2 The role of BMAs as key components of the bone marrow microenvironment in lung cancer bone metastasis

#### 4.2.1 The effect of BMAs on OCs in lung cancer bone metastasis

BMAs can secrete factors like RANKL, IL-6, and TNF-α, which activate OCs to promote bone resorption within the bone microenvironment (93, 94). *In vitro* studies have shown that BMAs can upregulate the RANK expression on OCs, leading to increased OC formation (95). Research on breast cancer bone metastasis demonstrated that activated OCs release acid phosphatase, acidifying the bone microenvironment (96, 97). This acidic environment upregulates matrix MMPs, and BMAs further contribute to bone matrix degradation by upregulating expression of OC-specific genes such as cathepsin K, facilitating tumor cell growth (98). IL-1β and IL-6 can induce EMT in breast cancer cells via the STAT3 pathway and promote angiogenesis, which suggests that BMAs may similarly exacerbate bone destruction in lung cancer bone metastasis (**Figure 2**).

#### 4.2.2 The effect of BMAs on OBs in lung cancer bone metastasis

Studies have shown that enhanced adipogenic differentiation of bone marrow MSCs in the bone marrow microenvironment leads to a decrease in their osteogenic potential (99). BMAs contribute to the regulation of OB function by secreting inflammatory factors such as IL-6, IL-1β, and TNF-α (100–102). Both tumor cells and BMAs can produce IL-6, which promotes tumor cell proliferation, induces OC activation, and downregulates OB activity (102). Additionally, IL-6 promotes adipogenic differentiation of MSCs while inhibiting osteogenesis (103). An increase in palmitic acid and arachidonic acid, associated with increased BMAs, heightens the cytotoxic effects on OBs. *In vitro* studies indicate that palmitate-induced lipotoxicity in OBs and osteocytes is mediated by autophagy dysregulation, leading to OB apoptosis (104). Given that lung cancer cells also secrete IL-6 and TNF-α, it is speculated that lung cancer cells and BMAs, by regulating OB function and inhibiting osteogenic differentiation, indirectly disrupt bone remodeling and promote lung cancer bone metastasis (**Figure 2**).

#### 4.2.3 The effect of BMAs on ECs in lung cancer bone metastasis

BMAs can regulate EC function through both direct and indirect mechanisms. Directly, they secrete cytokines and metabolic products influencing EC growth, proliferation, and migration (105–107). *In vitro* studies have found that adiponectin secreted by BMAs affects EC growth and migration and influences the expression of EC adhesion molecules, facilitating closer proximity and invasion by tumor cells into bone (107, 108). Indirectly, cytokines secreted by BMAs activate multiple signaling pathways affecting ECs. Studies on breast cancer bone metastasis showed that BMAs-secreted IL-1β activates the p38-MAPK pathway, increasing EC permeability and vasculogenesis (108). Additionally, the impact of BMAs-secreted IL-6 on HIF-1α and VEGF levels participates in the regulation of angiogenesis (109). The presence of adiponectin and IL-6 in the bone marrow microenvironment of lung cancer bone metastasis suggests that BMAs may promote EC growth and new blood vessel formation, providing additional nutrients and pathways for cancer metastasis (**Figure 2**).

#### 4.2.4 The role of inflammatory factors secreted by BMAs in lung cancer bone metastasis

BMAs are capable of secreting pro-inflammatory cytokines such as IL-1β, IL-6, TNF-α, and leptin, and they induce BMSCs to participate in the inflammatory immune response by regulating B cell responses and lymphocyte production (110). For instance, the inflammatory cytokine IL-1β, secreted by BMAs, can upregulate the expression of leptin (111). A clinical study involving 116 lung cancer patients with bone metastasis found significantly higher levels of leptin and its receptor in patients with bone metastases compared to those without, suggesting that the formation of lung adenocarcinoma bone metastatic lesions is closely related to leptin (112). Leptin can inhibit the activity of macrophages and natural killer cells, reducing their ability to kill lung cancer cells. It can also regulate the expression of immune checkpoint molecules on the surface of lung cancer cells, such as programmed death-ligand 1 (PD-L1), to inhibit the immune cells’ ability to kill tumor cells. PD-L1, in conjunction with its receptor PD-1, forms an immune checkpoint that suppresses the activation and proliferation of effector T cells and promotes the increase and upregulation of regulatory T cells (Tregs) (113, 114). Ultimately, lung cancer cells can escape immune surveillance by regulating the leptin signaling pathway, thereby promoting the occurrence and development of lung cancer bone metastases. Additionally, IL-6, TNF-α, CXCL12, and leptin are considered to significantly promote tumor cell migration and proliferation, as well as inhibit apoptosis and activate autophagy, facilitating the development of tumor bone metastasis (115, 116) (**Figure 2**).

#### 4.2.5 The role of BMAs as an energy source in lung cancer bone metastasis

Current research posits that BMAs, through their metabolic processes, produce a substantial quantity of lipids, including fatty acids, triglycerides, phospholipids, and cholesterol (117, 118). These lipids serve as an effective energy source for tumor cells, promoting their proliferation, migration, and invasion (118). Lipids are not only essential components of tumor cell membranes but also serve as energy sources during high metabolic demands (117). Fatty acids, fundamental components of lipids, have been found to be released from triacylglycerol in BMAs through lipolysis. These fatty acids can supply energy for tumor cell growth and metabolism via the microcirculation (119). Furthermore, fatty acid-binding protein 4 (FABP4) secreted by BMAs can increase the stability of fatty acids. FABP4 facilitates lipid transport and the transfer of free fatty acids to tumor cells, playing a role in the process of tumor bone metastasis (120, 121). Studies utilizing BMA-enriched mouse models found upregulated levels of FABP4 in Prostate Cancer(PCa) cells directly in contact with BMAs, suggesting a bidirectional interaction between FABP4 and the PPARγ pathway may enhance the invasiveness of tumor cells in bone metastasis (121). Additionally, co-culture studies inducing lipolysis in adipocytes and beta-oxidation in cancer cells have demonstrated that adipocytes can act as an energy source for cancer cells (122). In PCa, *in vitro* studies showed that adipocytes could enhance *PCa* cell migration. Breast cancer research found that adipocytes near invasive cancer cells promoted the migration and growth of breast tumor cells (119). These studies collectively underscore the significant role of BMAs as an energy source in the process of tumor bone metastasis (123). Given the metabolic secretion of a large amount of lipids by BMAs in the bone marrow microenvironment of lung cancer bone metastasis, it is speculated that BMAs, as an energy source, facilitate the development of lung cancer bone metastasis.

Furthermore, certain immunomodulatory adipokines secreted by BMAs may participate in the process of tumor bone metastasis through interactions with adipocytes, immune cells, and tumor cells (110, 124). The following sections will discuss the mechanisms of action of leptin, adiponectin, Nesfatin-1, Resistin, chemerin, and visfatin in lung cancer bone metastasis.

## 5 The action of adipokines secreted by BMAs in lung cancer bone metastasis

### 5.1 Leptin

Leptin is a protein composed of 146 amino acids encoded by the *ob* gene, acting as a neuromodulatory, immunoregulatory, and endocrine hormone with multifunctional roles across various organs (125). By binding to specific leptin receptors, leptin activates intracellular signaling pathways, regulating the transcription of target genes to exert biological effects (125). The interaction of leptin with central and peripheral receptors yields divergent, sometimes opposing effects; while binding to peripheral receptors may increase bone mass, interaction with central receptors can induce bone loss (126).

#### 5.1.1 Direct effects of leptin in lung cancer bone metastasis

The binding of leptin to its receptor can activate several signaling pathways associated with tumor progression, including TGF-β, JAK/STAT, PI3K, HIF, and MAPK pathways (127). *In vitro* studies on lung cancer bone metastasis have shown that leptin promotes metastasis of the A549 human lung cancer cell line through a TGF-β-dependent induction of EMT (128). Leptin can also block the endoplasmic reticulum stress-related pathway, preventing apoptosis and promoting proliferation in lung adenocarcinoma A549 cells (129). Inhibition of the leptin-related pathway significantly induces apoptosis in these cells. Ieptin can regulate apoptosis-related factors, such as members of the Bcl-2 family and caspases, to inhibit apoptosis in lung cancer cells (130). This evidence confirms that leptin enhances lung cancer cell growth and migration through suppressing apoptosis (**Table 1**).

**Table 1:** Leptin, Adiponectin, Nesfatin-1, Resistin, Chemerin, Visfatin: Mechanisms of action in tumor bone metastasis - Literature table.

BMAs	Tumor types	Promote or inhibit tumor bone metastasis	Impacts	Mechanisms of action	References
**Leptin**	Lung cancer	Promote	Promotion of tumor cell migration	Induction of EMT via TGF-β	(128)
			Promotion of tumor cell survival	Inhibition of endoplasmic reticulum stress and apoptosis	(129)
			Promotion of tumor cell survival	Regulation of apoptotic factors such as Bcl-2 family,cysteine proteases,inhibiting apoptosis	(130)
			Promotion of osteolysis	Increase in ICAM-1 expression,combination with NF-κB inducing OC formation	(131)
		inhibit	Inhibition of osteolysis	Inhibition of OB production of RANKL and inhibition of OC differentiation	(131)
			Inhibition of osteolysis	Up-regulation of OPG expression and hindering of RANKL/RANK binding	(131)
**Adiponectin**	Lung cancer	Promote	Promotion of tumor microenvironment angiogenesis	Regulation of VEGF expression and signaling pathways	(132, 133)
			Promotion of osteolysis	Direct action on OC to promote OC formation and activation	(134)
			Promotion of osteolysis	Regulation of OC differentiation through the RANK/RANKL, JAK/STAT, and MAPK signaling pathways	(135–139)
		inhibit	Inhibition of tumor proliferation	Regulation of OC differentiation through the PI3K/Akt and mTOR signaling pathways	(140)
			Promotion of tumor cell apoptosis	Induction of apoptosis by activating AMPK signaling to increase the BAX/Bcl-2 ratio	(141)
**Nesfatin-1**	Colon cancer	Promote	Promotion of tumor cell migration and invasion	Induction of EMT-associated proteins	(142)
**Resistin**	Lung cancer	Promote	Promotion of osteolysis	Activation of NF-κB signaling pathway to promote OC activation and function enhancement	(143)
			Promotion of osteolysis	Inhibition of OB differentiation and promotion of OC activation, leading to bone destruction	(144)
			Promotion of tumor microenvironment inflammation	Promotion of activation of immune inflammatory cells, regulation of cytokines, and affecting the tumor microenvironment	(145, 146)
			Promotion of tumor microenvironment angiogenesis	Activation of signals such as JAK/STAT and PI3K/AKT to promote angiogenesis	(146, 147)
**Chemerin**	Oral cancer	Promote	Promotion of angiogenesis	Recruitment of immune cells,promotion of angiogenesis, and regulation of bone remodeling through Wnt/β-catenin signaling	(148–150)
	Breast cancer	inhibit	Inhibition of osteolysis	Blocking of RANKL inducing OC formation	(151)
**Visfatin**	Chondrosarcoma	Promote	Promotion of tumor migration	Synthesis of MMPs-2 via pro-inflammatory signaling pathways	(152)

TGF-β, Transforming Growth Factor Beta; EMT, Epithelial-Mesenchymal Transition; Bcl-2, B-cell lymphoma 2; ICAM-1, Intercellular Adhesion Molecule 1; NF-κB, Nuclear Factor Kappa-Light-Chain-Enhancer of Activated B Cells; OC, Osteoclast; OB - Osteoblast; RANK, Receptor Activator of Nuclear Factor Kappa-B; RANKL, Receptor Activator of Nuclear Factor Kappa-B Ligand; OPG, Osteoprotegerin; VEGF, Vascular Endothelial Growth Factor; JAK/STAT, Janus kinase/signal transducer and activator of transcription; MAPK, Mitogen-activated protein kinase; PI3K/Akt, Phosphoinositide 3-kinase/protein kinase B; AMPK, AMP-activated protein kinase; BAX/Bcl-2 ,Bcl-2-associated X protein/B-cell lymphoma 2; Wnt/β-catenin, Wingless-related integration site/beta- catenin; MMPs-2, Matrix Metalloproteinases-2.

#### 5.1.2 Indirect effects of leptin in lung cancer bone metastasis

Leptin’s indirect effects in lung cancer bone metastasis mainly involve regulation of OCs and OBs. Leptin inhibits the expression of RANKL in OBs, thereby suppressing OC differentiation; it also increases the expression of OPG, preventing the RANKL/RANK binding and indirectly inhibiting bone resorption (131). Leptin can regulate other factors and pathways affecting bone resorption, such as matrix MMP2 and MMP9, which are involved in extracellular matrix remodeling, tumor progression, and bone absorption (131, 153). Clinical studies on lung cancer bone metastasis have found that overactivation of MMP2/MMP9 promotes osteolytic metastasis and bone destruction in advanced cancer (154). *In vitro* studies showed that leptin enhanced production of soluble intercellular adhesion molecule-1 (ICAM-1) in lung cancer cells through triggering a signaling cascade involving JAK1/2, STAT3, FAK, ERK, and GSK3αβ (154). Leptin-stimulated production of soluble ICAM-1, in coordination with RANKL activation, synergistically induces OC formation, suggesting that leptin indirectly promotes tumor-induced bone resorption. Animal experiments have shown that leptin promotes proliferation and differentiation of OBs and enhances bone matrix synthesis and secretion, including collagen, alkaline phosphatase, and osteocalcin (155, 156). Further research is needed to determine whether this effect is similarly active in lung cancer bone metastasis (**Table 1**).

#### 5.1.3 Immune modulation by leptin in lung cancer bone metastasis

Leptin directly influences the proliferation, differentiation, and activity of various immune cells (such as monocytes, T cells, B cells, and macrophages) and interacts with other cytokines in the tumor microenvironment (157). Leptin indirectly regulates immune function in the lung cancer bone metastasis microenvironment by affecting the function of other cells (158). Cell studies have demonstrated that leptin promotes monocyte proliferation, induces macrophage phagocytosis and pro-inflammatory cytokine secretion, and acts as a nutritional factor to prevent apoptosis, playing a role in adaptive immunity by regulating T and B cell populations (159). Leptin dose-dependently promotes naïve CD4+ T cell proliferation and polarizes CD4+ T cells towards a Th1 phenotype (160), which in turn facilitates lung cancer secretion of inflammatory cytokines such as TNF-α and IL-6, promoting lung cancer bone metastasis. Beyond its effects on T cells, leptin maintains the homeostasis of murine B cells by inducing Bcl-2 and cyclin D1, promoting cell cycle entry and preventing apoptosis, thereby promoting lung cancer cell proliferation (161). Leptin interacts with inflammatory cytokines, such as VEGF and TGF-β, to establish and maintain an inflammatory immune state within the tumor microenvironment (162). Leptin can also upregulate the function of MDSCs, which suppress T cell activation and proliferation through the release of immunosuppressive factors and direct interaction with T cells (163), allowing lung cancer cells to evade immune surveillance (**Table 1**).

It is crucial to note that the specific mechanisms of leptin in lung cancer bone metastasis require further investigation due to the complexity of tumor metastasis and individual variability, which may lead to diverse leptin response patterns. Additionally, factors such as lung cancer type (e.g., adenocarcinoma, squamous cell carcinoma), molecular subtypes, and the microenvironment may also influence the effects of leptin.

### 5.2 Adiponectin

While previous studies suggested adiponectin was primarily secreted by white adipose tissue (WAT), recent research shows that BMAs produce more adiponectin than WAT, especially in cancer patients. Adiponectin’s role in the tumor microenvironment contrasts with that of leptin, sparking debate over its impact on cancer (164). Although earlier results pointed towards anti-tumor effects, recent studies have highlighted adiponectin’s significant role in promoting tumor metastasis (165).

#### 5.2.1 Direct effects of adiponectin in lung cancer bone metastasis

Adiponectin primarily exerts its influence by inhibiting the proliferation of lung cancer cells and inducing their apoptosis. An *in vitro* study on lung cancer revealed that adiponectin can suppress the proliferation of lung cancer cells by inhibiting the PI3K/Akt signaling pathway and the phosphorylation of mTOR (mammalian target of rapamycin) (140). Additionally, it induces cell apoptosis by activating the AMPK signaling pathway and increasing the BAX/Bcl-2 ratio (140, 141) (**Table 1**). Animal experimental studies have also discovered adiponectin’s role in prohibiting lung cancer cell proliferation through inhibiting the Wnt/β-catenin signaling pathway (166).

#### 5.2.2 Indirect effects of adiponectin in lung cancer bone metastasis

Adiponectin indirectly participates in lung cancer bone metastasis by regulating angiogenesis, influencing OC activities, and impacting immune functions. Research indicates that adiponectin affects vessel formation by regulating the expression of VEGF and its signaling pathways (165). Adiponectin can enhance the expression of VEGFs (including VEGF-A, VEGF-B, VEGF-C, and VEGF-D) in ECs. VEGFs operate on receptors on ECs, such as VEGFR-1 and VEGFR-2, promoting EC proliferation and tubule formation through activating the VEGFR-2 signaling pathway. Moreover, adiponectin promotes angiogenesis by inhibiting angiogenesis inhibitors, such as Angiopoietin-1 (132, 133).

Adiponectin directly and indirectly partakes in the development and maturation of OCs, influencing bone metastasis in lung cancer. On one side, *in vitro* experiments have shown that adiponectin can directly influence OCs, promoting their formation, activation, and increasing the release of lysosomal enzymes, thereby enhancing bone resorption (134). On the other hand, it indirectly contributes to the formation and maturation of OCs mainly through binding to receptors on cell types such as OBs and OCs (167). Receptors include Ob-Rb (long-form receptor primarily distributed in MSCs); upon binding, adiponectin can activate multiple signaling pathways and regulate the expression of related genes, contributing to the differentiation of hematopoietic stem cells (HSCs) into OCs (166). Pathways implicated include RANK/RANKL, JAK/STAT, and MAPK, promoting bone metastasis by influencing OC formation and maturation (135–139) (**Table 1**). In summary, adiponectin exerts an indirect influence on osteolytic metastasis in lung cancer primarily through modulating angiogenesis within the tumor microenvironment, participating in the regulation of OC activity, and affecting the equilibrium of bone remodeling.

#### 5.2.3 Impact on immune regulation

Adiponectin might play a role in lung cancer bone metastasis through regulating immune cell functions. *In vitro* studies indicate that adiponectin can increase the quantity and function of regulatory T cells (Tregs) while reducing the activity of natural killer (NK) cells, diminishing attacks on cancer cells, thus creating a favorable environment for lung cancer bone metastasis (168). However, differing studies suggest adiponectin possesses anti-inflammatory properties, inhibiting the NF-κB signaling pathway and cytokine release, thereby reducing the production of inflammatory mediators. Consequently, it suppresses the inflammatory response surrounding lung cancer cells, reducing their survival and proliferation capabilities (169, 170).

### 5.3 Nesfatin-1

Discovered in 2006, Nesfatin-1 is an anorexigenic neuropeptide initially associated with food intake and energy regulation, hence being considered as a hormone regulating body weight and appetite. Beyond the central system, Nesfatin-1 is also present in various organs and tissues, such as the stomach, intestines, spleen, and adipose tissue (171). However, research on Nesfatin-1 secreted by BMAs in lung cancer bone metastasis is lacking. Recent studies have predominantly focused on Nesfatin-1 secreted by white adipose tissue, observing elevated expression levels of Nesfatin-1 in lung cancer, breast cancer, and other tumors. It has been found that Nesfatin-1 is associated with tumor invasion, metastasis, and prognosis (172, 173).

#### 5.3.1 Direct role of Nesfatin-1 in tumor bone metastasis

Nesfatin-1 secreted by adipose tissue in the tumor microenvironment might directly influence tumor bone metastasis by regulating the expression and function of EMT-related proteins like E-cadherin, N-cadherin, and Vimentin (174). Some studies have demonstrated that Nesfatin-1/Nucleobindin-2 can suppress the expression of E-cadherin and increase the expression of N-cadherin and Vimentin, thereby inducing migration, invasion, and EMT of colon cancer cells (142) (**Table 1**). Since both peripheral and bone marrow fat can secrete Nesfatin-1, it can be inferred that in the bone marrow microenvironment of lung cancer bone metastasis, Nesfatin-1 secreted by BMAs might promote the migration and invasion of lung cancer cells by inducing EMT.

#### 5.3.2 Indirect role of Nesfatin-1 in tumor bone metastasis

Nesfatin-1 might indirectly affect the invasion and metastasis of tumor cells in bone tissue by influencing the activity of immune cells and the production of inflammatory cytokines (175). Clinical research has shown that Nesfatin-1 can induce an increase in CCL2 expression in human synovial tissues, favoring M1 macrophage polarization, thereby increasing the expression of pro-inflammatory cytokines like IL-1β, IL-6, and TNF-α, and promoting the progression of inflammatory responses (176). This, consequently, indirectly affects the invasion and metastasis of tumor cells in bone tissue. Additionally, studies in rats have found that Nesfatin-1 can inhibit cell apoptosis induced by IL-1β inflammation and promote angiogenesis by inhibiting neutrophil recruitment, cell apoptosis, and activating VEGF, mechanisms that can indirectly promote tumor bone metastasis (177, 178). There is currently no direct evidence regarding Nesfatin-1’s mechanisms in lung cancer bone metastasis, further research to clarify these mechanisms is required.

### 5.4 Resistin

Resistin, a peptide hormone, is primarily secreted by white adipose tissue but also produced by other tissues such as bone marrow adipose, liver, and muscle (179). Initial studies suggested its association with insulin resistance and type 2 diabetes (180). Recent research, however, has linked Resistin to bone marrow fat and bone metabolism, suggesting it may influence OC and osteoblast OB functions, participate in inflammatory responses and immune regulation, and affect the tumor microenvironment in tumor bone metastasis.

#### 5.4.1 Direct role of resistin in lung cancer bone metastasis

Resistin may directly promote lung cancer bone metastasis by activating OC function. *In vitro* studies have revealed that resistin can activate the NF-κB signaling pathway, leading to the release of inflammatory cytokines like TNF-α and IL-6, which further enhance OC activation and functionality, accelerating bone tissue destruction and bone metastasis (143). Additionally, resistin has been found to inhibit OB differentiation and bone-forming functions while promoting OC activation and functionality, resulting in bone tissue destruction and remodeling (144) (**Table 1**).

#### 5.4.2 Indirect role of resistin in lung cancer bone metastasis

By regulating the production of inflammatory cytokines and the activity of immune cells, as well as activating tumor-associated signaling pathways like JAK/STAT and PI3K/AKT, resistin can indirectly promote cellular growth, angiogenesis, and immune response in the tumor microenvironment, facilitating lung cancer bone metastasis (145). Resistin can promote the production and activation of inflammatory cells like macrophages and lymphocytes and regulate the inflammatory response by modulating cytokine release (146) (**Table 1**). These immune cells play an essential role in lung cancer bone metastasis, including promoting inflammatory responses, regulating immune responses, and affecting tumor cell invasion and migration. Moreover, animal models of lung cancer have shown that mice treated with anti-resistin antibodies exhibited reduced rates of lung cancer development and metastasis (181), further proving that resistin, by modulating inflammatory responses in the tumor microenvironment, indirectly facilitates lung cancer bone metastasis.

Resistin can activate signaling pathways such as JAK/STAT and PI3K/AKT, thereby influencing cell growth and angiogenesis within the tumor microenvironment, as well as suppressing the body’s anti-tumor immune response, indirectly facilitating the occurrence and progression of tumor bone metastasis. Studies have found that resistin activates the PI3K and Akt signaling pathways, while inhibitors of PI3K and Akt, or siRNA, can reduce the expression of VEGF-A induced by resistin (146). Concurrently, both *in vitro* and *in vivo* studies have demonstrated that resistin promotes the expression of VEGF-A and angiogenesis within the tumor microenvironment by inhibiting the expression of miR-5-3p through the PI3K/Akt signaling cascade, thus affecting tumor bone metastasis (146, 147) (**Table 1**).

### 5.5 Chemerin

Chemerin is a small peptide hormone predominantly secreted by adipose tissue, with subsequent production noted in the liver, kidneys, and other tissues. Initially identified for its roles in inflammation and immune regulation, recent clinical studies have unveiled Chemerin’s capability to inhibit osteogenic differentiation in favor of adipogenesis (182). The Wnt pathway plays a crucial role in bone biology and the regulation of bone tumors, especially the classic Wnt/β-catenin signaling pathway is closely related to tumor bone metastasis. Relevant studies on mouse bone structure have shown that inhibiting the Wnt/β-catenin signaling pathway and activating the RANK signal promote bone resorption. Simultaneously, it has been demonstrated that Chemerin participates in the progression of bone tumors (151, 183). However, the role of Chemerin in cancer remains contentious, with a majority of studies highlighting its anticancer effects, while a minority suggest its protumorigenic capabilities. These effects derive from its regulation of angiogenesis and modulation of the immune-inflammatory response in the bone marrow microenvironment, indirectly contributing to the osteolytic metastasis of lung cancer.

Antitumor activities of Chemerin have been identified in a murine model of breast cancer, where it binds to its receptor ChemR23/CMKLR1 and inhibits neovascularization, thereby suppressing growth and invasion of breast cancer cells (148). Moreover, in Chemerin-treated media derived from metastatic breast cancer cells, an increase in the RANKL/OPG ratio and a reduction in the secretion of MMPs (e.g., MMP-2, MMP-9) and Cathepsin K were observed (148) (**Table 1**), thus inhibiting RANKL-induced OC formation and consequentially suppressing bone dissolution and tumor bone metastasis.

On the contrary, protumorigenic activity is evidenced through the recruitment of innate immune defenses and activation of endothelial vasculogenesis, as well as the suppression of Wnt/β-catenin signaling, reducing OB differentiation and stimulating OC differentiation and proliferation through RANK signaling activation (149). This mechanism plays a crucial role in tumor bone metastasis. Furthermore, studies in oral squamous cell carcinoma (OSCC) have shown that Chemerin enhances the formation of human umbilical vein ECs (HUVECs), promoting angiogenesis and subsequently tumor growth and migration. Additionally, Chemerin can upregulate pro-angiogenic factors such as VEGF-A, MMP-9, MMP-2, and S100A9 in neutrophils through the activation of the MEK/ERK signaling pathway (150, 184) (**Table 1**), thereby facilitating tumor vascularization and bone metastasis in lung cancer. Hence, the balance between Chemerin’s anticancer and protumorigenic effects ultimately dictates tumor progression, underscoring the need for further research into Chemerin’s role within the lung cancer bone metastasis.

### 5.6 Visfatin

Visfatin, an inflammatory adipokine also known as pre-B cell colony-enhancing factor (PBEF), plays a pivotal role in NAD+ biosynthesis as the rate-limiting enzyme in nicotinamide metabolism, implicating its involvement in B-cell development, apoptosis, and glucose metabolism. Initially discovered in visceral adipose tissue, Visfatin has been widely recognized for its compensatory response in obesity-induced insulin resistance (185). Recent studies have expanded our understanding of Visfatin’s functionality, uncovering its significant role in the genesis and development of tumors as well as bone metabolism. Elevated levels of Visfatin expression have been associated with certain tumor types, correlating with aggressiveness, migration, and prognosis (152). By promoting the synthesis of MMP-2 and activation of the AP-1 transcription factor through the ERK, p38, and JNK signaling pathways, Visfatin enhances tumor cell migration (186) (**Table 1**). Additionally, overexpression of Visfatin has been linked to increased pulmonary metastasis in a murine model of chondrosarcoma. In the mouse bone marrow, it disrupts the balance between bone resorption and formation, tilting towards a pro-inflammatory phenotype in MSCs differentiating towards BMAs and OBs (144). As an adipokine, Visfatin’s exact action in bone regulation and immune modulation remains to be fully elucidated. However, its capacity to induce pro-inflammatory transcription factors, such as NF-kB, and modulate pathways including MAPK, PI3K, and ROS (187), suggests that Visfatin might act as a potential tumor-promoting factor in lung cancer bone metastasis through upregulation of MMP-2, enhanced differentiation of OBs, and the promotion of inflammatory responses in MSCs, thereby indirectly facilitating osteolysis and the progression of pulmonary cancer bone metastasis.

## 6 Conclusion and future perspectives

This review primarily explored how BMAs directly or indirectly interact with various cells within the bone microenvironment of lung cancer, as well as its pathological role in modulating the tumor microenvironment and thus influencing bone metastasis in lung cancer. The contributions of BMAs are summarized through three main aspects: (1) BMAs provide direct energy supply to lung cancer cells through lipid metabolites and FABP4, with lipids also serving as building blocks for the cancer cell membrane; (2) Direct effects of BMAs in bone metastasis of lung cancer include secretion of pro-inflammatory cytokines like IL-1β that participate in PMN formation and CCL12 which directly interacts with CXCR4 on lung cancer cells, promoting cell proliferation and invasion. Secreted adipokines like leptin activate TGF-β, PI3K/Akt, and MAPK signaling pathways, upregulating MMP2/MMP9 secretion and facilitating EMT, thereby promoting bone metastasis; (3) Indirect effects of BMAs are mediated through interactions with OCs, OBs, ECs, and MSCs, indirectly aiding lung cancer bone metastasis. For instance, leptin secreted by BMAs may regulate osteogenesis and osteolysis by inhibiting OC activity, suppressing RANKL production and enhancing OPG secretion. Adiponectin influences OC activity, increases regulatory T cells, and inhibits NK cell activity, contributing to immune evasion by cancer cells. Resistin could activate the NF-κB pathway, releasing inflammatory cytokines like TNF-α and IL-6, thus promoting OC activation and inhibiting OB differentiation, essential for lung cancer bone metastasis.

Despite the existence of both metastasis-promoting and inhibitory factors secreted by BMAs, they predominantly exert a pro-metastatic role in lung cancer bone metastasis. Reducing BMAs in the bone microenvironment could represent a novel approach to inhibit lung cancer bone metastasis. Considering the shared origin and mutual regulation during differentiation between BMAs and OBs, promoting osteogenic differentiation to decrease BMAs might suppress lung cancer bone metastasis. Z-DNA binding protein 1 (ZBP1) has been identified as a novel regulator of osteogenic and adipogenic differentiation through the Wnt/β-catenin signaling pathway (188). Therefore, ZBP1 could serve as a novel therapeutic target for treating lung cancer bone metastasis. Additionally, inhibiting the actions of BMA-secreted factors could indirectly repress lung cancer bone metastasis. For instance, clinical research on metastatic renal cell carcinoma (RCC) patients undergoing tyrosine kinase inhibitor (TKI) therapy revealed that the adiponectin-AdipoR1 axis inhibits tumor cell migration and invasion by blocking the GSK3β/β-Catenin pathway (189). This raises the possibility that adiponectin-AdipoR1 might exhibit similar effects in lung cancer bone metastasis, potentially serving as a therapeutic target for further verification.

In summary, BMAs and their secreted adipokines present effective therapeutic targets for lung cancer bone metastasis, paving new directions for future research in this field.

## Author contributions

JL: Writing – original draft, Writing – review & editing. JW: Writing – original draft. YN: Writing – review & editing. XY: Writing – review & editing.
